# Predictors of measles-rubella vaccination status in the Savannah Region, Ghana: A cross-sectional study among caregivers of children aged 18–59 months

**DOI:** 10.1016/j.jvacx.2024.100567

**Published:** 2024-09-29

**Authors:** Michael Rockson Adjei, Kwabena Adjei Sarfo, Cyril Kwami Azornu, Peter Gyamfi Kwarteng, Felix Osei-Sarpong, Janet Vanessa Baafi, Byrite Asamoah, Chrysantus Kubio, Martin Peter Grobusch, Sally-Ann Ohene

**Affiliations:** a Center of Tropical Medicine and Travel Medicine, Department of Infectious Diseases, Amsterdam University Medical Centers, Location AMC, University of Amsterdam, Amsterdam, The Netherlands; b World Health Organization, Country Office, Accra, Ghana; c Ghana Health Service, Regional Health Directorate, Damongo, Savannah Region, Ghana; d Ghana Health Service, East Gonja Municipal Hospital, Salaga, Savannah Region, Ghana; e United Nations Children’s Fund, Ghana; f Ghana Health Service, District Health Directorate, Odumase, Sunyani West, Ghana; g Synlab, Accra, Ghana; h Institute of Tropical Medicine, and German Center of Infectious Diseases (DZIF), University of Tuebingen, Tuebingen, Germany; i Institute of Infectious Diseases and Molecular Medicine, University of Cape Town, Cape Town, South Africa; j Centre de Recherches Médicales en Lambaréné (CERMEL), Lambaréné, Gabon; k Masanga Medical Research Unit, Masanga, Sierra Leone

**Keywords:** Adverse event following immunization, Measles-rubella vaccine, Bole District, Central Gonja District, Savannah Region, Ghana

## Abstract

**Introduction:**

Savannah Region witnessed a decline in measles-rubella (MR) vaccination coverage prior to the measles outbreak in 2022. This study aimed to assess contributory factors of the low routine MR vaccination coverage and proffer recommendations to improve vaccination uptake.

**Methods:**

A cross-sectional study was conducted in two districts (Bole and Central Gonja) of Savannah Region from December 2022 to June 2023. Caregivers of children 18–59 months were randomly selected and interviewed using a structured questionnaire. Bivariate and multivariate logistic regression were performed to assess predictors of MR vaccination status.

**Results:**

Children of caregivers with inadequate knowledge of MR vaccination (AOR = 0.58, 95 %CI: 0.47–0.72), travelled more than five km to access health services (AOR = 0.48, 95 %CI: 0.39–0.59), described health workers attitude as poor (AOR = 0.44, 95 %CI: 0.26–0.74), and those who sought treatment for adverse events following immunization (AEFI) from the pharmacy (AOR = 0.65, 95 %CI: 0.51–0.84) were less likely to complete MR vaccination. On the contrary, children of female sex (AOR = 1.27, 95 %CI: 1.05–1.53), aged 24–59 month (AOR = 2.56, 95 %CI: 1.05–1.53), caregivers with primary or secondary education (AOR = 1.43, 95 %CI: 1.11–1.84; and AOR = 2.23, 95 %CI: 1.64–3.03 respectively), and those who did not experience rescheduling of vaccination sessions (AOR = 1.61, 95 % CI: 1.25–2.01) were more likely to complete routine MR vaccination schedule.

**Conclusion:**

Inadequate caregiver knowledge, poor geographical access to health services, poor healthcare worker attitude, and non-institutional management of AEFI significantly contributed to the low MR vaccination uptake in the Savannah Region. Adopting tailored approaches to addressing these factors could improve vaccination coverage.

## Introduction

Measles, a potentially deadly disease, is caused by a highly contagious virus that primarily spreads through respiratory droplets. The virus is capable of surviving in the air and on surfaces for an extended period, contributing to its high attack rate and case fatality [1], [2]. The disease remains a significant public health challenge worldwide, and children under five years are most at risk with approximately 142,000 deaths reported globally in 2018 [1], [2].

Efforts to eliminate measles, including vaccination, have been a priority at both global and regional levels [3]. The global measles elimination effort had veered off track prior to the COVID-19 pandemic. The declining immunization coverage was worsened by the COVID-19 pandemic, with the proportion of unvaccinated children increasing globally by 21 % between 2019 and 2021 [3]. As in some other world regions, including high-income settings in North America and across Europe [4]; many countries in Africa fell short of the measles elimination target of less than one case per 1,000,000; and as of 2021, the regional incidence stood at 81.9/1,000,000 [5]. By January 2022, cases had spiked by 400 % and countries including Ethiopia, Somalia, and Democratic Republic of Congo were among the worst-afflicted [6].

Measles has been a major cause of child mortality in Ghana. In 1977, the Ghana Health Assessment Project ranked measles second to malaria in terms of burden of disease, accounting for 7.3 % of the healthy days of life lost through illness, disability, and death [7]. Ghana has made significant strides against measles, having reduced the case burden by 72 % from 82,684 in 1980 to 23,068 in 2000. This improvement has largely been attributed to the steady increase in measles vaccination coverage from 24 % to 84 % within the same period [8], [9].

The impact of the pandemic on Ghana’s immunization programme was worsened by intermittent vaccine stockouts leading to many children missing measles-rubella (MR) vaccination [10]. The drop in population immunity contributed to sporadic measles outbreaks across the country: 88 cases (incidence: 2.7/1,000,000) were recorded in 2020; 1,274 (incidence: 38.8/1,000,000) in 2021; 395 (incidence: 11.8/1,000,000) in 2022; and as of the 5th epidemiological week in 2023, 50 new cases (incidence: 1.5/1,000,000) had been confirmed from 11 districts [11].

**Fig. 1:**
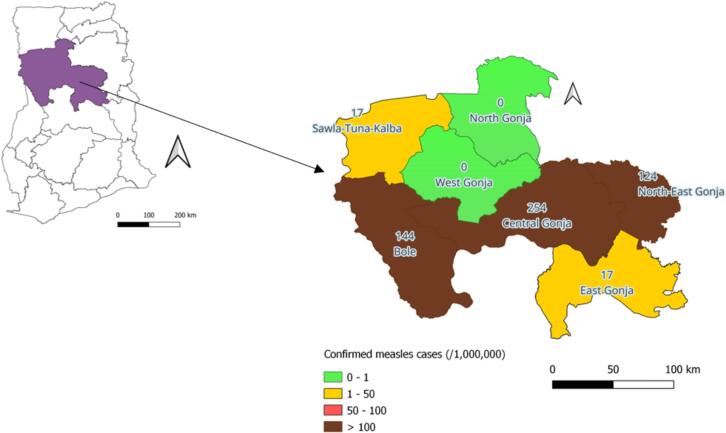
Confirmed measles incidence by district, Savannah Region, 2022. In set: Ghana map.

**Fig. 2:**
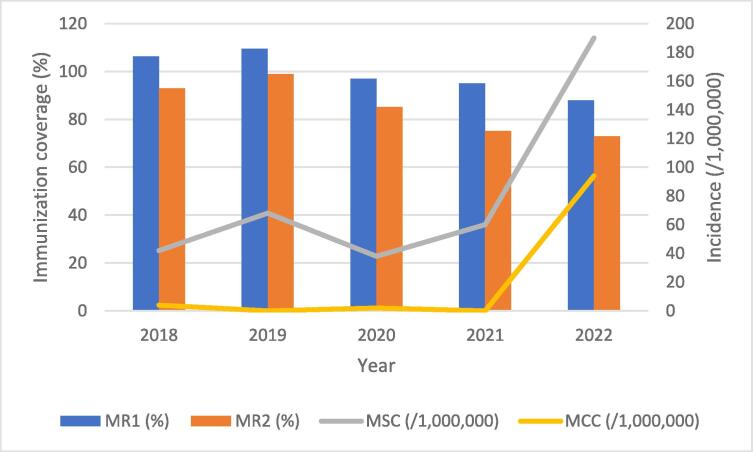
Measles-Rubella vaccination coverage and measles incidence, Savannah Region, Ghana; 2018–2022 (MSC: Suspected measles case; MCC: Confirmed measles cases).

The Savannah Region recorded surges in measles cases from 2020, and in 2022 the region was caught up in an outbreak involving all districts. A total of 63 (94/1,000,000 population) cases were confirmed with the majority (85.7 %) from Bole (26.9 %; 17/63 cases; 144/1,000,000) and Central Gonja (58.7 %; 37/63 cases; 254/1,000,000) districts, respectively (Fig. 1). Prior to the outbreak, measles-rubella (MR) vaccination coverage had declined sequentially with increasing dropout rates between the first (MR1) and second (MR2) doses of MR vaccine (Fig. 2). Although the region had intermittent stockout of MR vaccine (especially in 2022), uptake did not improve significantly during the periods of vaccine availability.

Demand- and supply-side factors have been found to impact uptake of MR vaccination. Caregiver education level is important because educated caregivers are more likely to remember dates; understand the importance of vaccination; or may have a higher income to afford transport and other fees [12], [13]. However, the impact of caregiver education on MR vaccination uptake could vary by level or setting. While Kantner et al. (2021) found secondary education to be an enabler [14], Danso et al. (2023) observed high caregiver education level to militate against MR vaccination uptake among children [15]. The age and sex of a child have also been found to influence the uptake of MR vaccination. Research conducted by Kassahun et al. (2015) in Northwest Ethiopia found that male children were more likely to be vaccinated compared with female children [16]. Kantner et al. (2021) found that the older the child, the greater the likelihood of being fully vaccinated [14].

Although previous studies conducted into factors associated with MR vaccination may provide valuable insight, the applicability of the findings may be context dependent, hence the need for the current study. This study aimed to assess the contributory factors of low routine MR vaccination uptake in the Savannah Region and proffer recommendations to increase coverage and improve population immunity to mitigate future outbreaks.

## Methods

### Study design

A cross-sectional study was conducted in the Savannah Region from December 2022 to June 2023.

### Study setting

The Savannah Region was carved out from the Northern Region in 2019. It is bordered in the north by the Upper West and North East regions, in the west by Côte d'Ivoire and Burkina Faso, in the south by the Bono and Bono East regions, and in the east by Northern and Oti regions (Fig. 1). It is the region with the largest land size in Ghana and occupies an area of 35,862 km^2^ with a total population of 653,266 [17]. The region is arid, with one rainy season occurring typically annually between July and October. The majority of the inhabitants are peasant farmers pursuing grain farming and livestock rearing; most of the livestock farmers are undocumented nomads from neighbouring countries [18].

There are seven administrative districts (Fig. 1) and 179 healthcare facilities comprising five hospitals, three polyclinics, 23 health centres, and 148 community-based health planning and services (CHPS) compounds. Most (>50 %) of the communities are hard-to-reach and located more than five km from the nearest health facility [18]. There is a generally poor road network, and access to communities off the main road network is worsened during the rainy season (July to October) due to springing-up of water bodies leading to flooding, which affects health service delivery. Immunization services are provided through a fixed strategy (facility-based vaccination sessions), and mobile strategy (community-to-community vaccination sessions) across the region.

Bole District is in the south-western part of the region and shares an international boundary with Côte d’Ivoire. The district is one of the region’s economic hubs and serves as a transit point for some travellers in the West African sub-region. The total population, according to the 2021 population and housing census, is 115,800 with an annual growth rate of 3.1 % [17]. There are seven subdistricts, 186 communities, and 32 health facilities comprising a district hospital, a polyclinic, two clinics, seven health centres, and 21 CHPS compounds.

Central Gonja District stretches from the eastern towards the central part of the region and shares a border with Bole. It is the most-industrialized district in the region and serves as transit point for commuters to Burkina Faso and other West African countries. The total population is 142,762 according [17], and there are six subdistricts, 272 communities, and 48 health facilities including a district hospital, a polyclinic, four health centres, and 31 CHPS compounds. Central Gonja is drained by the Black and White Volta rivers and some of its communities are difficult to access especially during rainy seasons [18].

### Data collection

Epi Info statistics software (Epi Info version 7.2.2.16, https://www.cdc.gov>epiinfo) was used to estimate the sample sizes for the respective districts based on the following parameters: expected proportion of children completing MR vaccination – 66 % (Ghana Demographic and Health Survey, 2022); margin of error – 5 %; and confidence level – 95 %. The minimum sample sizes were 337 and 338 for Bole and Central Gonja districts, respectively. However, because the study was conducted in response to the measles outbreak, it was integrated with active case search for measles, and a greater number of caregivers were interviewed.

Forty-five health staff comprising disease surveillance officers and community health nurses were recruited as data collectors. A two-day training was conducted for the data collectors at the regional capital, Damongo by regional level officers. The training content included application of measles case definition, sampling technique, conducting active case search, informed consenting process, and administration of the data collection tools. The data collection tools were piloted in Atributo, a community in Damongo and finalized.

A multi-stage cluster sampling was employed, and communities (clusters) were selected by probability proportionate to size, ensuring representation of each subdistrict. An average of five caregivers were interviewed in each selected community, i.e., one per household. A caregiver was defined as any person 16 years or above with primary responsibility for the health care of a child aged 18–59 months. In each cluster, the first household was selected from a random direction from the centre of the community by spinning a pen. Subsequent households were selected to the right of the first, skipping two houses between selections.

In the households, children were randomly selected, and individual written informed consents were obtained from the caregivers (respondents) before administration of the questionnaire. Privacy was ensured and the interviews were carried out using an electronic questionnaire on Kobo Collect (Kobo Toolbox, https://www.kobotoolbox.org/), – an open-source data collection application.

MR vaccination status was validated with information from home-based vaccination records. Sociodemographic characteristics (including age of respondent, sex, average monthly income, education level, parity/number of children if biological caregiver, and marital status); knowledge of MR vaccination schedule (adequate knowledge was defined as knowing that two routine doses of MR given at nine and 18 months were required for complete vaccination status; otherwise, inadequate knowledge); and service experience (including postponement or otherwise of vaccination sessions; health worker attitude towards service delivery – good or poor; and experience of adverse events following immunization) were collected. Data was synced to a central server at the end of each interview.

### Data analysis

Data was extracted onto Microsoft Excel 16.0 spreadsheet and exported into Epi Info statistical software (Epi Info version 7.2.2.16, https://www.cdc.gov>epiinfo) for analysis. Descriptive statistics (mean, median, standard deviation, range, first and third quartiles) were estimated for age (caregivers and children). Frequency and percentage distribution of characteristics were computed with cross-tabulations to compare MR vaccination status dichotomized as complete (received two doses) and incomplete (received only one dose or none). Independent variables that showed significant association with the outcomes (p < 0.05) were included in the logistic regression model to assess determinants of a child’s MR vaccination status. The results were presented as odd ratio at 95 % confidence level.

## Results

A total of 2,189 respondents comprising 997/2,189 (45.5 %) and 1,192/2,189 (54.5 %) from Bole and Central Gonja districts, respectively, participated in the study. Approximately 56 % (1,219/2,189) of the children sampled (Bole – 669/997, 67.1 %; Central Gonja – 550/1,192, 46.2 %) had completed routine MR vaccination (two doses); about 18 % (402/2,189) had received only one routine dose (Bole – 155/997, 15.5 %; Central Gonja – 20.7 %, 247/1,192); and nearly 26 % (568/2,189) had received no routine dose (Bole – 173/997, 17.4 %; Central Gonja – 395/1,192, 33.1 %).

The mean age of the respondents was 33.6 years, with a standard deviation of 10.3 years. The youngest respondent was 16 years old, while the oldest was 71 years. Twenty-five percent (first quartile, QI) were aged up to 26 years. Approximately 50 % (1,095/2,189) of the respondents (median) were 32 years old or below, while 1,642/2,189, 75 % (third quartile, Q3) were up to 39 years old. The majority (73.4 %, 1,606/2,189) were aged 20–39 years. The mean age of the children was 32.4 months (SD = 10.9; median = 32; Q1 = 23; Q3 = 38; range = 18–59). Most (74.1 %, 1,621/2,189) of them were aged 24–59 months and majority (53.5 %, 1,172/2,189) was male. Approximately 60 % (1,324/2,189) of the respondents were biological parents, and 81 % (1,073/1,324) had at least three children. Only 22.2 % (486/2,189) of the participants were male, and 18.7 % (409/2,189) were itinerant (nomadic) residents. Approximately 63 % (1,370/2,189) had no formal education, and about 90 % (1,961/2,189) earned an average monthly income of less than 100 US dollars (Table 1A).

**Table 1:** (A) Characteristics of respondents, MR vaccination status, Savannah Region; 2022–2023. (B) Characteristics of respondents, MR vaccination status, Savannah Region; 2022–2023.

(A)				
Characteristic	MR vaccination status		Total N* (%)	p-value
	Complete n (%)	Incomplete n (%)		
***Background***				
**Age of caregiver (years)**				
<20	35 (2.9)	30 (3.1)	65 (3.0)	0.000
20–29	422 (34.6)	383 (39.5)	805 (36.8)	
30–39	443 (36.3)	358 (36.5)	801 (36.6)	
40–49	256 (21.0)	121 (12.5)	377 (17.2)	
≥50	63 (5.2)	78 (8.0)	141 (6.4)	

**Sex of caregiver**				
Male	287 (23.5)	199 (20.5)	486 (22.2)	0.100
Female	932 (76.5)	771 (79.5)	1703 (77.8)	

**Place of residence**				
Urban	1032 (84.7)	790 (81.4)	1822 (83.2)	0.052
Rural	187 (15.3)	180 (18.6)	367 (16.8)	

**Resident type**				
Itinerant (Nomad)	238 (19.5)	171 (17.6)	409 (18.7)	0.282
Established (Permanent)	981 (80.5)	799 (82.4)	1780 (81.3)	

**Education level (Caregiver)**				
No formal education	668 (56.4)	682 (70.3)	1370 (62.6)	0.000
Primary	250 (20.5)	174 (17.9)	424 (19.4)	
Secondary	239 (16.6)	85 (8.8)	324 (14.8)	
Tertiary	42 (3.5)	29 (3.0)	71 (3.2)	

**Marital status**				
Married/Cohabiting	131 (10.8)	154 (15.9)	285 (13.0)	0.000
Single/Divorced/Widowed	1088 (89.2)	816 (84.1)	1904 (87.0)	

**Age of child (months)**				
18–23	245 (44.7)	323 (55.3)	568 (25.9)	0.000
24–59	975 (60.1)	646 (39.9)	1621(74.1)	

**Sex of child**				
Male	629 (53.7)	543 (46.3)	1172 (53.5)	0.046
Female	590 (58.0)	427 (42.0)	1017 (46.5)	

**Parity (N = 1324)**				
1–2	148 (19.9)	103 (17.7)	251 (19.0)	0.523
3–4	422 (56.8)	346 (59.6)	768 (58.0	
≥5	173 (23.3)	132 (22.7)	305 (23.0)	

**Average monthly income ($)**				
<100	1052 (86.3)	909 (93.7)	1961 (89.6)	0.000
100–200	158 (13)	56 (5.8)	214 (9.8)	
≥200	9 (0.7)	5 (0.5)	14 (0.6)	
				

(B)				
Characteristic	MR vaccination status		Total N* (%)	p-value
	Complete n (%)	Incomplete n (%)		
***Knowledge about MR vaccine***				
**Informed about MR vaccine**				
Yes	1068 (56.3)	737 (76.0)	1805 (82.5)	0.000
No	151 (12.4)	233 (24.0)	384 (17.5)	

**Adequate knowledge (N = 1,805)**				
Yes	673 (63)	385 (52.2)	1058 (58.6)	0.000
No	395 (37)	352 (47.8)	747 (41.4)	

***Service Experience***				
**Community immunization services**				
Yes	119 (9.8)	153 (15.7)	272 (12.4)	0.000
No	32 (2.6)	80 (8.3)	112 (5.1)	
'Don't know'	1068 (87.6)	737 (76.0)	1805 (82.5)	

**Distance to nearest health facility**				
<5 km	895 (73.4)	539 (55.6)	1434 (65.5)	0.000
≥5 km	324 (26.6)	431 (44.4)	755 (34.5)	

**Postponement of vaccination session in the last three months (Service reliability)**				
Yes	187 (15.3)	181 (18.7)	368 (16.8)	0.000
No	1032 (84.7)	789 (81.3)	1821 (83.2)	

**Health worker attitude**				
Good	1193 (97.9)	911 (93.9)	2104 (96.1)	0.000
Poor	26 (2.1)	59 (6.1)	85 (3.9)	

**Place of AEFI management**				
Health facility	389 (31.9)	304 (31.3)	693 (31.7)	0.000
Pharmacy	247 (20.3)	303 (31.3)	550 (25.1)	
Home	583 (47.8)	363 (37.4)	946 (43.2)	

* N = 2189 unless otherwise stated.

**Fig. 3:**
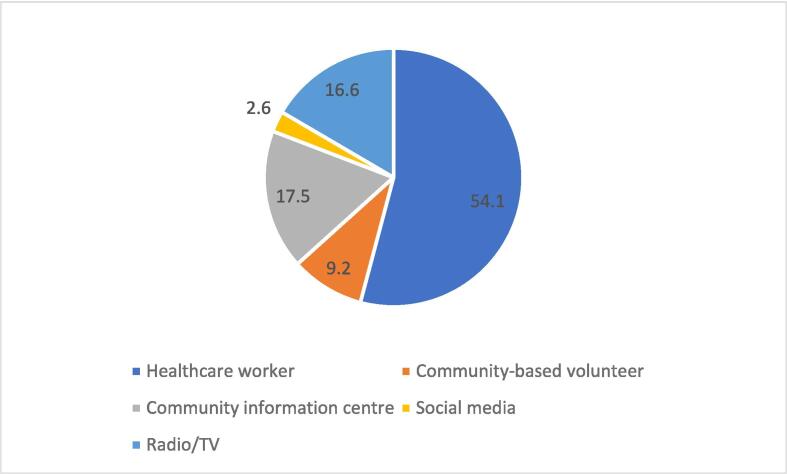
Respondents’ source of information about MR vaccine, Savannah Region; 2022–2023.

**Fig. 4:**
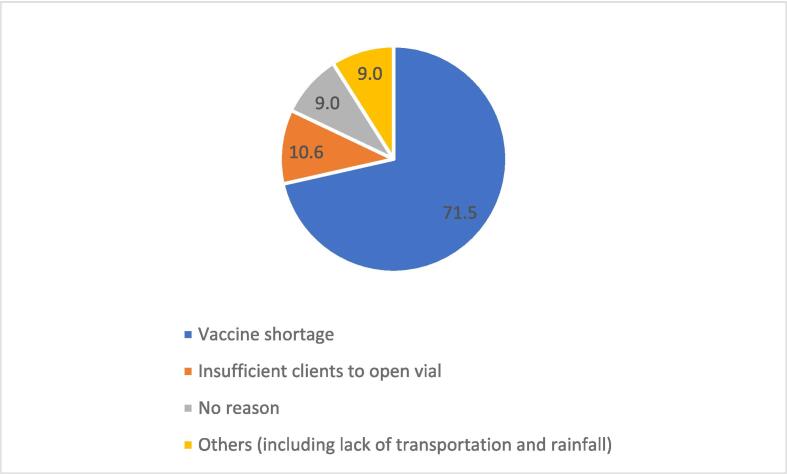
Reasons for postponement of vaccination session, Savannah Region; 2022–2023.

Nearly 83 % (1,805/2,189) were informed about MR vaccination, and 54.1 % (977/2,189) received the information from healthcare workers (Fig. 3). Of those that were informed about MR vaccination, only 58.6 % (1,058/1,805) had adequate knowledge. A greater proportion (82.5 %; 1,805/2,189) was unsure whether immunization services were provided in their community or the adjoining, while 34.5 % (755/2,189) travelled more than five km to the nearest health facility to access services. Approximately 17 % (368/2,189) of the respondents confirmed postponement of their child’s vaccination by healthcare workers and the main reason was stockout of vaccine (Fig. 4), but the majority (96.1 %, 2,104/2,189) was satisfied with the attitude of healthcare workers. All the respondents stated their children had ever experienced adverse event following immunization (AEFI). Approximately 90 % (1,970/2,189) of the AEFI were non-serious (constitutional symptoms and signs including fever, loss of appetite, diarrhoea, vomiting, among others) that resolved spontaneously or upon administration of over-the-counter medication (25.1 %, 550/2,189) or homemade (43.2 %, 946/2,189) remedies (Table 1B).

Multivariate logistic regression analyses showed that caregivers with primary or secondary education (AOR = 1.43, 95 %CI: 1.11–1.84; and AOR = 2.23, 95 %CI: 1.64–3.03), children aged 24–59 month (AOR = 2.56, 95 %CI: 1.05–1.53), children of female sex (AOR = 1.27, 95 %CI: 1.05–1.53), incomes of $100–200 (AOR = 3.41, 95 %CI: 2.32–5.02), and those that did not experience rescheduling of vaccination sessions in the preceding three months of the study (AOR = 1.61, 95 %CI: 1.25–2.01) were more likely to have children with complete MR vaccination status. Caregivers with tertiary education (AOR = 0.37, 95 %CI: 0.20–0.66), aged 50 years or more (AOR = 0.41, 95 %CI: 0.21–0.80), had inadequate knowledge of the MR vaccination schedule (AOR = 0.58, 95 %CI: 0.47–0.72), travelled more than five km to access health services (AOR = 0.48, 95 %CI: 0.39–0.59), described health workers attitude as poor (AOR = 0.44, 95 %CI: 0.26–0.74) and those who sought AEFI treatment from the pharmacy (AOR = 0.65, 95 %CI: 0.51–0.84) were less likely to have children with complete MR vaccination status. There was no significant association between access to community immunization services and complete MR vaccination status (Table 2).

**Table 2:** Estimated odds ratio of complete MR vaccination status, Savannah Region; 2022–2023.

Characteristic	OR	95 % CI	p-value	AOR	95 % CI	p-value
**Age of caregiver (years)**						
<20	1.0			1.0		
20–29	0.94	0.57–1.58	0.825	0.67	0.38–1.19	0.172
30–39	1.06	0.64–1.76	0.820	0.75	0.42–1.33	0.319
40–49	1.81	1.06–3.1	0.029	1.07	0.58–1.98	0.833
≥50	0.70	0.40–1.25	0.222	0.41	0.21–0.80	0.009

**Education level (Caregiver)**						
No formal education	1.0			1.0		
Primary	1.42	1.14–1.78	0.002	1.43	1.11–1.84	0.005
Secondary	2.79	2.13–3.65	0.000	2.23	1.64–3.03	0.000
Tertiary	1.44	0.89–2.33	0.144	0.37	0.20–0.68	0.002

**Marital status**						
Married/Cohabiting	1.0			1.0		
Single/Divorced/Widowed	1.57	1.22–2.01	0.000	1.30	0.98–1.74	0.072

**Age of child (months)**						
18–23	1.0			1.0		
24–59	1.98	1.64–2.41	0.000	2.56	2.05–3.18	0.000

**Sex of child**						
Male	1.0			1.0		
Female	1.91	1.01–1.41	0.41	1.27	1.05–1.53	0.012

**Average monthly income ($)**						
<100	1.0			1.0		
100–200	2.44	1.77–3.35	0.000	3.41	2.32–5.02	0.000
≥200	1.56	0.52–4.66	0.43	3.19	0.96–10.56	0.058

**Informed about MR vaccine**						
Yes	1.0			1.0		
No	0.45	0.36–0.56	0.000	0.82	0.61–1.11	0.200

**Adequate knowledge (N = 1805)**						
Yes	1.0			1.0		
No	0.52	0.44–0.62	0.000	0.58	0.47–0.72	0.000

**Access to community immunization services**						
Yes	1.0			1.0		
No	0.51	0.32–0.83	0.006	0.51	0.39–0.86	0.11
'Don't know'	1.86	1.44–2.41	0.000	1.0		

**Distance to nearest health facility**						
<5 km	1.0			1.0		
≥5 km	0.45	0.38–0.54	0.000	0.48	0.39–0.59	0.000

**Postponement of vaccination session in the last three months**						
Yes	1.0			1.0		
No	1.27	1.01–1.58	0.039	1.61	1.25–2.01	0.000

**Health worker attitude**						
Good	1.0			1.0		
Poor	0.34	0.21–0.54	0.000	0.44	0.26–0.74	0.002

**Place of AEFI management**						
Health facility	1.0			1.0		
Pharmacy	0.64	0.51–0.80	0.000	0.65	0.51–0.84	0.001
Home	1.26	1.03–1.53	0.025	1.09	0.86–1.37	0.458

## Discussion

The study assessed predictors of MR vaccination status among children 18–59 months in the epicentres of measles outbreak in the Savannah Region of Ghana. Two doses of MR vaccine given at nine and 18 months, respectively, or soon after, are required for complete vaccination to confer adequate protection against the disease [19]. Our study found that only 55.7 % of the children had complete vaccination status. This proportion was less than the projection of 66.4 % from the 2022 Ghana Demographic and Health Survey (GDHS) [20], although both estimates were lower than the administrative coverage of 72.9 % [Savannah Regional Health Directorate, Annual Report 2022, unpublished]. The differences in the estimates might lie in the methods: While our study focused on children aged 18–59 months, the GDHS estimated MR coverage among children aged 24–35 months. Again, our study used home-based immunization records as the only proof of vaccination, but the GDHS confirmed vaccination status from home-based immunization record or caregiver recall. Given that immunization records retention could be as low as 24 % in some settings [21], its use as sole proof of vaccination status might result in underestimation of vaccination coverage.

The relatively low MR coverage in Central Gonja (46.1 %) was probably due to poor access to services especially among riverine communities [18]. Nonetheless, the coverage difference was of little significance given that both districts were below the herd immunity threshold and were affected to similar extent by the measles outbreak. Averting or mitigating measles outbreaks requires population immunity coverage of at least 95 % (through vaccination or natural infection) [19].

Our study found that caregivers with primary or secondary level education were more likely to fully vaccinate their children against measles, and this was corroborated by the findings of Kantner et al. (2021) [14]. The observation that caregivers with tertiary education were less likely to have children with complete MR vaccination status was consistent with the findings of Danso et al. (2023) [15]. While higher education may be associated with general hesitancy to vaccination [22], [23], our study did not assess the reasons for the low MR uptake among children of caregivers with tertiary education.

While the 2022 GDHS reported no significant difference in MR uptake among the sexes [20], our study found that female children were more likely to be fully vaccinated compared with male, contrary to the observation of Kassahun et al. (2015) [16]. The differences in observations might lie in the methodology. While our study focused on only MR vaccination, Kassahun et al. (2015) assessed the vaccination status of children based on administration of multiple childhood vaccines including measles, bacillus Calmette–Guerin (BCG), and diphtheria-tetanus-pertussis (DPT)-containing vaccines. On the other hand, although the 2022 GDHS provided coverages for individual vaccines at the subnational level, gender analysis was reported only at the national level, and the finding may not be generalizable. The gender influence on MR vaccination status as observed from our study might be attributed to the effect of sociocultural factors on immunization uptake. In patriarchal societies, male children spend more time with their fathers – being nurtured to take up gender roles – and considering that caregiver role often falls to women [24], they are more likely to miss vaccinations compared with female children.

We found that older children (24–59 months) were more likely to be fully vaccinated compared with those aged 18–23 month, and this aligned with the findings of Kantner et al. (2021) [14]. According to the vaccination schedule, a child in Ghana completes MR vaccination by 18 months and those missing appointments could be vaccinated up to 59 months [20]. The high proportion of children vaccinated not before age 24 months is concerning, given that delays increase the risk of contracting severe measles infection.

Healthcare workers are perceived as ambassadors of the healthcare system [25]; therefore, gaps in their knowledge and attitude could damage the immunization programme. Our study observed that caregivers who considered the attitude of healthcare workers as poor were less likely to have children with complete MR vaccination status. According to Asamani et al. (2017) high workload and low job satisfaction were major contributors to poor attitude among healthcare workers in Ghana [26].

Caregivers who sought treatment from the pharmacy for AEFI were less likely to have children with complete MR vaccination status. The guideline for AEFI management in Ghana is not explicit on payment for treatment [27], although anecdotal evidence suggests the existence of an unwritten policy for management at the nearest public health facility at no cost to the caregiver or client. Again, although AEFI treatment is covered by Ghana’s national health insurance scheme [27], persons without valid registration may have to pay out of pocket including other ancillary costs. It is possible that caregivers who sought AEFI treatment from the pharmacy did so to avoid cost associated with facility-based management, and such experience could impact the uptake of vaccination in the future.

A limitation of the study was that respondents’ recall of information that were not available from the home-based immunization records (for example average monthly income, postponement of vaccination session, opinion on healthcare worker attitude, etc.) might have been affected due to psychological trauma, especially among those who were directly impacted by the outbreak. Mental health is affected during outbreaks and other emergency situations; and emotional disturbances, stress, depression, mood alterations, irritability, insomnia, post-traumatic stress disorder, anger, and emotional exhaustion could have consequences on recall capacity [28]. However, this was mitigated by repeating and rephrasing of questions to validate responses.

Additionally, it is possible the findings might not be representative of the entire Savannah Region due to selection of respondents from only two out of seven districts. However, given the similarity of profile to the other districts, and consistency of the findings with those of studies conducted under similar settings, the observations are reasonably generalizable.

Further research is needed to understand the low MR vaccination uptake among caregivers with high education.

## Conclusion

The proportion of children with complete MR vaccination in the epicentres of the 2022 measles outbreak in the Savannah Region was lower than the administrative coverage. Factors including inadequate caregiver knowledge, poor geographical access to health services, poor healthcare worker attitude, and non-institutional management of AEFI significantly contributed to the low MR vaccination uptake in the Savannah Region.

The Savannah Regional Health Directorate should support the districts to conduct periodic in-service training for healthcare workers on quality immunization service delivery including client-centred care to improve service experience and uptake. Healthcare workers should strengthen public education on the benefits and schedule of MR vaccination at every contact with caregivers, deploying interpersonal or mass communication approaches, where appropriate. Targeted approaches to reminding caregivers of vaccination due dates including the use of short message services (SMS) and new (social) media should be explored by health facilities to improve continuity of vaccination uptake. While steps are taken to establish additional health facilities in the long-term, the district health directorates should increase the frequency and coverage outreach services to reduce the distance travelled by caregivers to access immunization services; this is of particular importance for the riverine communities and other hard-to-reach areas, especially in Central Gonja District. Health facilities should strengthen point of care screening of immunization records to identify and vaccinate missed children. Lastly, the Ghana Health Service should consider strengthening the policy on AEFI management, ensuring cases are treated at health facilities at no cost to vaccinees or health facilities. The National Health Insurance Authority (NHIA) should consider reimbursing the cost of treatment to health facilities, even in situations where the vaccinee is not an active member of the scheme. Implementing these strategies would improve the coverage of measles vaccination to bridge the immunity gap and mitigate future outbreaks.

## Ethical approval statement

Ethical clearance was sought from the Ghana Health Service Ethics Review Committee (ID number: GHS-ERC 002/10/23). Administrative permission was obtained from the Savannah Regional Health Directorate for the use of institutional data. Individual written informed consent was sought from the respondents. Participant names and/or identifiers were anonymized to ensure privacy and confidentiality. The study data were stored on a computer protected with a password and used specifically for the study.

## Funding

This research did not receive any specific grant from funding agencies in the public, commercial, or not-for-profit sectors.

## Disclaimer

MRA and SAO are affiliated to the World Health Organization. The authors alone are responsible for the views expressed in this publication, and they do not necessarily represent the views, decisions, or policies of the World Health Organization.

## CRediT authorship contribution statement

**Michael Rockson Adjei:** Writing – review & editing, Writing – original draft, Methodology, Investigation, Formal analysis, Data curation, Conceptualization. **Kwabena Adjei Sarfo:** Methodology, Investigation. **Cyril Kwami Azornu:** Methodology, Investigation. **Peter Gyamfi Kwarteng:** Writing – review & editing, Data curation. **Felix Osei-Sarpong:** Methodology, Conceptualization. **Janet Vanessa Baafi:** Methodology, Investigation, Formal analysis. **Byrite Asamoah:** Validation, Methodology, Investigation. **Chrysantus Kubio:** Writing – review & editing, Writing – original draft, Methodology, Conceptualization. **Martin Peter Grobusch:** Writing – review & editing, Supervision. **Sally-Ann Ohene:** Writing – review & editing, Validation, Supervision.

## Declaration of competing interest

The authors declare that they have no known competing financial interests or personal relationships that could have appeared to influence the work reported in this paper.

## Data Availability

Data will be made available on request.
