# Supplementary material for: Predictors of measles-rubella vaccination status in the Savannah Region, Ghana: A cross-sectional study among caregivers of children aged 18–59 months
Source: Vaccine X. 2024 Sep 29;20:100567. doi: 10.1016/j.jvacx.2024.100567 (PMC11474206; doi:10.1016/j.jvacx.2024.100567)
Supplement: Supplementary Data 1 [file mmc1.docx]

**INSTRUCTIONS:** Randomly sample 15-20 households (HHs) per day for three (3) days. Randomly select and interview one caregiver of a child aged 18-59 months per household

**General Background Information**

1. Name of District
   1. Bole
   2. Central Gonja
2. Sub-district Name
3. Name of Volunteers
4. Telephone Number
5. Name of Community
6. GPS Coordinate
7. Name of CHPS

**MEASLES-RUBELLA SECOND DOSE DEFAULTER RATE AMONG CHILDREN 18 – 59 MONTHS**

1. Age of respondent
2. Sex
   1. Male
   2. Female
3. Place of residence
   1. Urban
   2. Rural
4. Settlement type
   1. Nomadic
   2. Non-nomadic
5. Ethnic group
   1. Gonja
   2. Dagomba
   3. Kamara
   4. Tampulma
   5. Dagaaba/Waala
   6. Vagla
   7. Fulani
   8. Mamprusi
   9. Other
6. 6. Religion
   1. Christianity
   2. Islam
   3. African Traditional Religion
   4. Other
7. Highest level of education
   1. No formal education
   2. Primary school
   3. Secondary
   4. Tertiary
8. Marital status
   1. Single
   2. Married/Cohabitation
   3. Widowed/Divorced
9. Occupation
   1. Unemployed
   2. Government worker
   3. Private sector worker
   4. Sel employed
   5. Farmer
   6. Trader
   7. Retired from formal employment
   8. Other
10. Average monthly income
    1. <$100
    2. $100-200
    3. >200

**Knowledge of Caregivers on Measles-Rubella Vaccine**

1. Relationship with child
   1. Mother
   2. Father
   3. Sibling (Brother/Sister)
   4. Aunt/Uncle
   5. Cousin/Niece/Nephew
2. Parity (Skip this question if not parent)
3. Have you ever heard of measles-rubella vaccine?
   1. Yes
   2. No
4. If yes, where did you hear it from?
   1. Television
   2. Radio
   3. Health staff
   4. Social media
   5. Community members (colleagues)
   6. Other (please specify where you heard it from)
5. How many times is a child supposed to take the measles-rubella vaccine?
   1. Once
   2. Twice
   3. I don’t know

1. At what age are children required to receive the measles-rubella vaccine?
   1. 9 months
   2. 18 months
   3. 9 and 18 months
   4. I don’t know

**Health System Factors on Measles Rubella Vaccine Uptake**

1. Do health workers come to your community for immunization services?
   1. Yes
   2. No
2. When was the last time health staff visited your community for immunization?
3. How long does it take to travel from your community to the nearest health facility?
   1. <5km
   2. ≥5km
4. Have health workers cancelled scheduled immunization sessions in the past three (3) months
   1. Yes
   2. No

If yes, what was the reason given for the cancellation of the scheduled vaccination session

1. Have health workers told you about the shortage of vaccine the last time your child was due for measles-rubella vaccination?
   1. Yes
   2. No
2. How will you describe the attitude of health staff who attend to you at CWC during vaccination?
   1. Good
   2. Poor
3. Does the attitude of health staff negatively influence the vaccination uptake of your child?
   1. Yes
   2. No

If yes, in which of the following ways do attitude of health staff affect the measles rubella vaccine uptake of your child

1. The health workers do not tell me when to come for the vaccine

ii. The health workers do not explain to me the specific ages that my child is supposed to take the vaccine

iii. The poor human relationship that health workers exhibit at the CWC scares me from sending my child for the vaccination

iv. Other (please specify attitude of health staff

1. Do you have experience with any adverse effects of the measles-rubella vaccine in your child?
   1. Yes
   2. No
2. If there were any adverse effects of the MR vaccine, which of the following describes the experience that affects the measles-rubella vaccine uptake of your child?
   1. My child became sick after taking the vaccine
   2. My child developed an abscess after taking the vaccine
   3. My child lost appetite after taking the vaccine
   4. Other (please specify adverse effects)
3. How did you handle the adverse reaction of your child?
   1. I took the child to a health facility for treatment
   2. I bought medicine from Pharmacy/Chemical shop for the child
   3. I applied cold compress on the arm
   4. Other (please specify how did you handle the adverse reaction)

**Demographic Information of Child and Observation for MR Vaccine Uptake From MCHRB**

1. Age (in months)
2. Sex
   1. Male
   2. Female
3. Place of birth
   1. Health Facility
   2. Home/TBA

**Measles Rubella Vaccine Uptake**

1. Has the child taken MR 1
   1. Yes
   2. No
2. MR1

Date

1. MR2

Date
